# Data glove-based gesture recognition using CNN-BiLSTM model with attention mechanism

**DOI:** 10.1371/journal.pone.0294174

**Published:** 2023-11-17

**Authors:** Jiawei Wu, Peng Ren, Boming Song, Ran Zhang, Chen Zhao, Xiao Zhang

**Affiliations:** 1 School of Medical Information and Engineering, Xuzhou Medical University, Xuzhou, China; 2 Engineering Research Center of Medical and Health Sensing Technology, Xuzhou Medical University, Xuzhou, China; University of Southampton - Malaysia Campus, MALAYSIA

## Abstract

As a novel form of human machine interaction (HMI), hand gesture recognition (HGR) has garnered extensive attention and research. The majority of HGR studies are based on visual systems, inevitably encountering challenges such as depth and occlusion. On the contrary, data gloves can facilitate data collection with minimal interference in complex environments, thus becoming a research focus in fields such as medical simulation and virtual reality. To explore the application of data gloves in dynamic gesture recognition, this paper proposes a data glove-based dynamic gesture recognition model called the Attention-based CNN-BiLSTM Network (A-CBLN). In A-CBLN, the convolutional neural network (CNN) is employed to capture local features, while the bidirectional long short-term memory (BiLSTM) is used to extract contextual temporal features of gesture data. By utilizing attention mechanisms to allocate weights to gesture features, the model enhances its understanding of different gesture meanings, thereby improving recognition accuracy. We selected seven dynamic gestures as research targets and recruited 32 subjects for participation. Experimental results demonstrate that A-CBLN effectively addresses the challenge of dynamic gesture recognition, outperforming existing models and achieving optimal gesture recognition performance, with the accuracy of 95.05% and precision of 95.43% on the test dataset.

## 1. Introduction

With the rapid development of computer technology and artificial intelligence, Human Machine Interaction (HMI) has emerged as one of the most prominent research fields in contemporary times. The driving force behind HMI is our expectation that machines will become intelligent and perceptive like humans [1]. HMI refers to the process of exchanging information between humans and machines through effective dialogue. HMI systems can collect human-intended information and transform it into a format understandable by machines, enabling machines to operate based on human intent [2]. Traditional HMI primarily relies on tools such as joysticks, keyboards, and mice to control terminals, which usually require fixed operational spaces. This severely restricts the range of human expressive actions and diminishes work efficiency. Consequently, to enhance the naturalness of HMI, the next generation of HMI technology needs to be human-centric, diversified, and intelligent [3]. In real-life situations, besides verbal communication, gestures serve as one of the most significant means for humans to convey information, enabling direct and effective expression of user needs. Research conducted by Liu et al. pointed out that hand gestures constitute a significant part of human communication, with advantages including high flexibility and rich meaning, making them an important modality in HMI [4]. Consequently, Hand Gesture Recognition (HGR) has emerged as a new type of HMI technology and has become a research hotspot with enormous potential in various domains. For instance, in the healthcare domain, capturing and analyzing physiological characteristics related to finger movements can significantly assist in studying and developing appropriate rehabilitation postures [5]. In the field of mechanical automation, interaction between fingers and machines can be achieved by detecting finger motion trajectories [6]. In the field of virtual reality, defining different gesture commands allows users to control the movements of virtual characters from a first-person perspective [7].

Research on HGR can be classified into two categories based on the methods of acquiring gesture data: vision-based HGR and wearable device-based HGR. Vision-based HGR relies on cameras as the primary tools for capturing gesture data. They offer advantages such as low cost and no direct contact with the human hands. However, despite the success of high-quality cameras, vision-based systems still have some inherent limitations, including a restricted field of view and high computational costs [8, 9]. In certain scenarios, robust results may require the combined data acquisition from multiple cameras due to issues like depth and occlusion [10, 11]. Consequently, the presence of these aforementioned challenges often hinders vision-based HGR methods from achieving optimal performance. In recent years, wearable device-based HGR has witnessed rapid development due to advancements in sensing technology and widespread sensor applications. Compared to vision-based approaches, wearable device-based HGR eliminates the need to consider camera distribution and is less susceptible to external environmental factors such as lighting, occlusion, and background interference. Data gloves represent a typical example of wearable devices used in HGR. These gloves are equipped with position tracking sensors that enable real-time capture of spatial motion trajectory information of users’ hand postures. Based on predefined algorithms, gesture actions can be recognized, mapped to corresponding response modules, and thus complete the HMI process. HGR systems based on data gloves have become a research hotspot in the relevant field. These systems offer several advantages, including stable acquisition of gesture data, reduced interference from complex environments and satisfactory modeling and recognition results, especially when dealing with large-scale gesture data [12].

In the field of HGR, researchers primarily focus on two types of gestures: static gestures and dynamic gestures. Static HGR systems analyze hand posture data at a specific moment to determine its corresponding meaning. However, static gesture data only provide spatial information of hand postures at each moment, while temporal information of hand movements is disregarded. As a result, the actual semantic information conveyed is limited, making it challenging to extend to complex real-world applications. Dynamic HGR systems, on the other hand, deal with information regarding the changes in hand movement postures over a period of time. These systems require a comprehensive consideration of both spatial and temporal aspects of hand postures. Clearly, compared to static gestures, dynamic gestures can convey richer semantic information and better align with people’s actual needs in real-life scenarios. Although numerous research efforts have been dedicated to dynamic HGR algorithms, most are based on vision systems, and the challenge of dynamic HGR using data gloves remains.

The dynamic gesture investigated in this study is the seven-step handwashing, which is a crucial step in the healthcare field. Proper handwashing procedures can effectively reduce the probability of disease transmission. Our work applies the seven-step handwashing to medical simulation training, where users wear data gloves to perform the handwashing process. Additionally, we design an automated dynamic gesture recognition algorithm to assess whether users correctly execute the specified hand gesture steps. Specifically, we developed a data glove-based dynamic HGR algorithm in this paper by incorporating deep learning techniques. This algorithm considers both spatial and temporal information of gesture data. Firstly, the Convolutional Neural Network (CNN) is utilized to extract local features of gesture data at each moment. Subsequently, these features are incorporated into the Bidirectional Long Short-Term Memory (BiLSTM) structure to model the temporal relationships. Finally, an attention mechanism is employed to enhance the gesture features and output the recognition results of dynamic gestures. In summary, this paper makes three main contributions:

1. Within the context of medical simulation, a data glove-based seven-step handwashing dynamic hand gesture data collection process was defined, and dynamic hand gesture data from 32 subjects were collected following this procedure.
2. A novel data glove-based dynamic HGR algorithm, called Attention-based CNN-BiLSTM Network (A-CBLN), was designed by combining deep learning techniques with the characteristics of dynamic gesture data. A-CBLN integrates the advantages of CNN and BiLSTM, effectively capturing the spatiotemporal features of gesture data, and further enhancing the features using an attention mechanism, resulting in precise recognition of dynamic gestures.
3. Extensive experiments were conducted to verify the effectiveness of the A-CBLN algorithm for dynamic gesture recognition, and key parameter settings within A-CBLN were thoroughly discussed. The results obtained from the test dataset demonstrated that our proposed method outperformed other comparative algorithms in terms of accuracy, precision, recall and F1-score.

The remaining sections of this paper are organized as follows. In Section 2, we review recent works related to HGR, with a particular focus on data glove-based HGR methods. Section 3 provides a detailed description of the proposed algorithm for dynamic gesture recognition. Section 4 encompasses the data collection methodology for gestures and provides implementation details of the conducted experiments. The relevant experimental results and analysis are presented in Section 5, followed by a concise summary of this paper in Section 6.

## 2. Related works

In recent years, research in the HGR field has focused on two main aspects: the type of gesture data (static or dynamic) and the sensors used for data collection (visual systems or wearable devices). This section provides an overview of relevant studies in HGR, emphasizing research involving wearable devices like data gloves.

Static hand gesture recognition research primarily focuses on analyzing the spatial features of gesture data without considering its temporal variations. This type of research is primarily applied in sign language recognition scenarios. A static hand gesture recognition system based on wavelet transform and neural networks was proposed by Karami et al. [13]. The system operated by taking hand gesture images acquired by a camera as input and extracting image features using Discrete Wavelet Transform (DWT). These features were fed into a neural network for classification. In the experimental section, 32 Persian sign language (PSL) letter symbols were selected for investigation. The training was conducted on 416 images, while testing was performed on 224 images, resulting in a test accuracy of 83.03%. Thalange et al. [14] introduced two novel feature extraction techniques, Combined Orientation Histogram and Statistical (COHST) Features and Wavelet Features, to address the recognition of static symbols representing numbers 0 to 9 in American Sign Language. Hand gesture data was collected using a 5-megapixel network camera and processed with different feature extraction methods before input into a neural network for training. The proposed approach achieved an outstanding average recognition rate of 98.17%. Moreover, a novel data glove with 14 sensor units was proposed by Wu et al. [15], who explored its performance in static hand gesture recognition. They defined 10 static hand gestures representing digits 0–9 and collected data from 10 subjects, with 50% of the data used for training and the remaining 50% for testing. By employing a neural network for classification experiments, they achieved an impressive overall recognition accuracy of 98.8%. Lee et al. [16] introduced a knitted glove capable of pattern recognition for hand poses and designed a novel CNN model for hand gesture classification experiments. The experimental results demonstrated that the proposed CNN structure effectively recognized 10 static hand gestures, with classification accuracies ranging from 79% to 97% for different gestures and an average accuracy of 89.5%. However, they only recruited 10 subjects for the experiments. Antillon et al. [17] developed an intelligent diving glove capable of recognizing 13 static hand gestures for underwater communication. They employed five classical machine learning classification algorithms and conducted training on hand gesture data from 24 subjects, with testing performed on an independent group of 10 subjects. The experimental results indicated that all classification algorithms achieved satisfactory hand gesture recognition performance in dry environments, with accuracies ranging from 95% to 98%. The performance slightly declined in underwater experimental conditions, with accuracies ranging from 81% to 94%. Yuan et al. [18] developed a wearable gesture recognition system that can simultaneously recognize ten types of numeric gestures and nine types of complex gestures. They utilized the Multilayer Perceptron (MLP) algorithm to recognize 19 static gestures with 100% accuracy, showcasing the strong capabilities of deep learning technology in the field of HGR. However, it is worth noting that the sample data in their experimental section was derived solely from four male volunteers. Moreover, a data glove based on flexible sensors was utilized by Ge et al. [19] to accurately predict the final hand gesture before the completion of the user’s hand movement in real time. They constructed a gesture dataset called Flex-Gesture, which consisted of 16 common gestures, each comprising 3000 six-dimensional flexion data points. Additionally, they proposed a multimodal data feature fusion approach and employed a combination of neural networks and support vector machines (SVM) as classifiers. The system achieved a remarkable prediction accuracy of 98.29% with a prediction time of only 0.2329 ms. However, it should be noted that the data glove-based system had certain limitations as it did not consider temporal information in the hand gestures. It is worth mentioning that the authors believe that incorporating deep learning algorithms with temporal features analysis could potentially yield more effective results.

Unlike static gesture recognition, dynamic gesture recognition requires considering the spatial information of hand movements and their temporal variations. With the rapid advancement of deep learning techniques, researchers have extensively investigated structures such as Convolutional Neural Network (CNN) and Recurrent Neural Network (RNN) and applied them to real-time dynamic gesture recognition problems. Nguyen et al. [20] presented a novel approach for continuous dynamic gesture recognition using RGB video input. Their method comprises two main components: a gesture localization module and a gesture classification module. The former aims to separate gestures using a BiLSTM network to segment continuous gesture sequences. The latter aims to classify gestures and efficiently combine data from multiple channels, including RGB, optical flow, and 3D key pose positions, using two 3D CNNs and a Long Short-Term Memory (LSTM). The method was evaluated on three publicly available datasets, achieving an average Jaccard index of 0.5535. Furthermore, Paweł et al. [21] developed a system capable of rapidly and effectively recognizing hand gestures in hand-body language using a dedicated glove with ten sensors. Their experiments defined 22 hand-body language gestures and recorded 2200 gesture data samples (10 participants, each gesture action repeated 10 times). Three machine learning classifiers were employed for training and testing, resulting in a high sensitivity rate of 98.32%. The pioneering work of Emmanuel et al. [22] introduced the use of CNN for grasp classification using piezoelectric data gloves. Experimental data were collected from five participants, each performing 30 object grasps following Schlesinger’s classification method. The results demonstrated that the CNN architecture achieved the highest classification accuracy (88.27%). It is worth mentioning that the authors plan to leverage the strengths of both CNN and RNN in future work to improve gesture prediction accuracy. Lee et al. [23] developed a real-time dynamic gesture recognition data glove. They employed neural network structures such as LSTM, fully connected layers, and novel gesture localization and recognition algorithms. This allowed the successful classification of 11 dynamic finger gestures with a gesture recognition time of less than 12 ms. Yuan et al. [24] designed a data glove equipped with 3D flexible sensors and two wristbands and proposed a novel deep feature fusion network to capture fine-grained gesture information. They first fused multi-sensor data using a CNN structure with residual connections and then modeled long-range dependencies of complex gestures using LSTM. Experimental results demonstrated the effectiveness of this approach in classifying complex hand movements, achieving a maximum precision of 99.3% on the American Sign Language dataset. Wang et al. [25] combined attention mechanism with BiLSTM and designed a deep learning algorithm capable of effectively recognizing 10 types of dynamic gestures. Their proposed method achieved an accuracy of 98.3% on the test dataset, showing a 14.5% improvement compared to a standalone LSTM model. This indicates that incorporating attention mechanism can effectively enhance the model’s understanding of gesture semantics. Dong et al. [12] introduced a novel dynamic gesture recognition algorithm called DGDL-GR. Built upon deep learning, this algorithm combined CNN and temporal convolutional networks (TCN) to simultaneously extract temporal and spatial features of hand movements. They defined 10 gestures according to relevant standards and recruited 20 participants for testing. The experimental results demonstrated that DGDL-GR achieved the highest recognition accuracy (0.9869), surpassing state-of-the-art algorithms such as CNN and LSTM. Hu et al. [26] explored deep learning-based gesture recognition using surface electromyography (sEMG) signals and proposed a hybrid CNN and RNN structure with attention mechanism. In this framework, CNN was employed for feature extraction from sEMG signals, while RNN was utilized for modeling the temporal sequence of the signals. Experimental results on multiple publicly available datasets revealed that the performance of the hybrid CNN-RNN structure was superior to individual CNN and RNN modules.

Despite the existence of a large body of research on HGR, research on dynamic gesture recognition using data gloves is still limited, especially in exploring the feasibility of applying deep learning in this field. Therefore, this study focused on the intelligent recognition of handwashing steps in the context of medical simulation. We utilized data gloves as the medium for dynamic gesture data collection and selected the seven-step handwashing series of dynamic gestures as the research target. Specifically, we considered the characteristics of dynamic gestures, including local feature variations in spatial positions and temporal changes in sequences. We systematically combined structures such as CNN, BiLSTM, and attention mechanism and designed a deep learning algorithm for dynamic gesture recognition based on data gloves. The next section will provide a detailed introduction to the proposed algorithm framework.

## 3. Methodology

### 3.1. Convolutional neural network (CNN)

A classic CNN architecture was designed by LeCun et al. in 1998 [27], which achieved remarkable performance in handwritten digit recognition tasks. Compared to traditional neural network structures, CNN exhibits characteristics of local connectivity and weight sharing [28]. Consequently, CNN can improve the learning efficiency of neural networks and effectively avoid overfitting issues caused by excessive parameters. The classic CNN architecture consists of three components: the convolutional layer, the pooling layer, and the fully connected layer.

The convolutional layer’s core component is the convolutional kernel (or weight matrix). Each convolutional kernel multiplies and sums the corresponding receptive field elements in the input data. This operation is repeated by sliding the kernel with a certain stride on the input data until the entire data has been processed for feature extraction. Finally, these feature maps are typically generated as the output of the convolutional layer through a non-linear activation function. It is worth mentioning that multiple convolutional kernels are usually chosen to extract more diverse features since each kernel extracts different feature information. ReLU [29] is the most popular activation function in CNN, it has the capability to retain the segments of input features that are greater than 0 and rectify the remaining segments to 0.

The pooling layer, also known as the down-sampling layer, extracts the minor features of the input data using pooling kernels. Similar to the convolutional kernels, each pooling kernel slides over the input data with a certain stride, preserving either the maximum value or the average value of the elements within the corresponding receptive field. This process continues until the feature extraction of the entire data is completed. The pooling layer is typically placed after the convolutional layers to reduce the dimensionality of the feature maps, thereby reducing the computational complexity of the entire network.

In classification tasks, the input data undergoes feature extraction by passing through multiple convolutional and pooling layers, and the resulting feature maps are flattened and fed into the fully connected layer. The fully connected layer usually consists of a few hidden layers and a softmax classifier, which further extracts features from the data and outputs the probability distribution of each class.

### 3.2. Bidirectional long short-term memory (BiLSTM)

The RNN is a recursively connected neural network with the ability of short-term memory that has been widely applied in the analysis and prediction of time series data [30]. However, due to memory and information storage limitations, RNN faces challenges in effectively learning long-term dependencies in time sequences, and gradient vanishing is often encountered during training [31]. To overcome these challenges, Greff et al. proposed the LSTM network structure that exhibits long-range memory capabilities [32]. The LSTM structure achieves this by introducing memory cells to retain long-term historical information and employing different gate mechanisms to regulate the flow of information. In fact, gate mechanisms can be understood as a multi-level feature selection approach. Consequently, compared to RNN, LSTM offer more advantages in handling time series problems.

**Fig 1:**
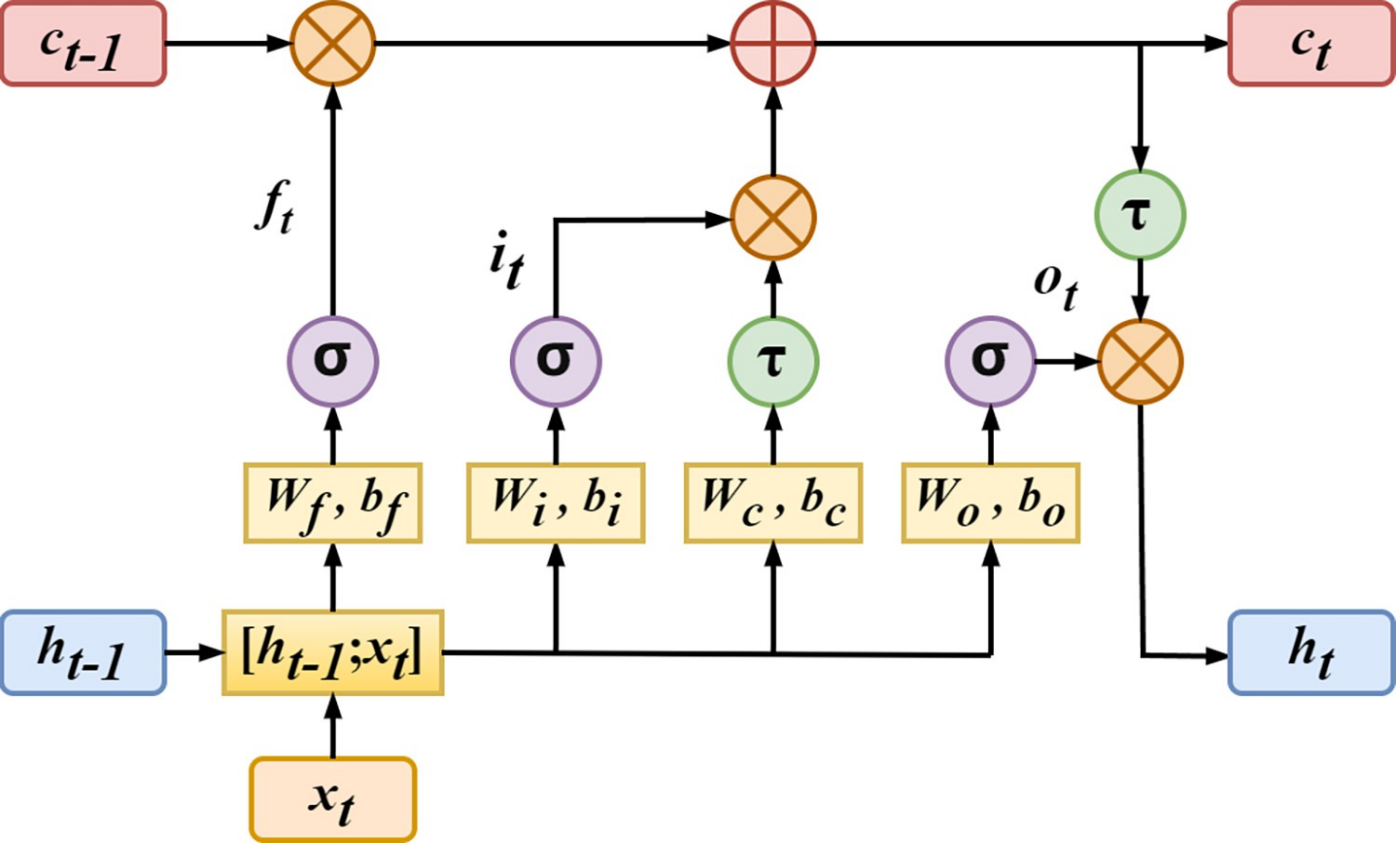
Internal structure of the LTSM unit.

The classical LSTM unit is equipped with three gate functions to control the state of the memory cell, denoted as the forget gate *f*_*t*_, input gate *i*_*t*_ and output gate *o*_*t*_. The forget gate *f*_*t*_ determines which information should be retained from the previous cell state *c*_*t*−1_ to the current cell state *c*_*t*_. The input gate *i*_*t*_ regulates the amount of information from the current input *x*_*t*_ that should be stored in the current cell state *c*_*t*_. The output gate *o*_*t*_ governs the amount of information from the current cell state *c*_*t*_ that should be transmitted to the current hidden state *h*_*t*_. Fig 1 illustrates the internal structure of a LSTM unit.

The LSTM unit has three inputs at time t: the current input *x*_*t*_, the previous hidden state *h*_*t*−1_, and the previous cell state *c*_*t*−1_. After being regulated by the gate functions, two outputs are obtained: the current hidden state *h*_*t*_ and the current cell state *c*_*t*_. Specifically, the output of *f*_*t*_ is obtained by linearly transforming the current input *x*_*t*_ and the previous hidden state *h*_*t*−1_, followed by the application of the sigmoid activation function. This process can be expressed by Formula 1.


$$f_{t}=\sigma (w_{f}*[h_{t-1},x_{t}]+b_{f})$$ (1)

Here, the weight matrix and bias vector of *f*_*t*_ are represented by *w*_*f*_ and *b*_*f*_, respectively. The sigmoid activation function, denoted by *σ*, is applied. The value of *f*_*t*_ ranges from 0 to 1, where a value closer to 0 indicates that information will be discarded, and a value closer to 1 implies more information will be preserved. The computation process of the input gate *i*_*t*_ is similar to that of *f*_*t*_, and the specific formula is as follows.


$$i_{t}=\sigma (w_{i}*[h_{t-1},x_{t}]+b_{i})$$ (2)

Here, the weight matrix and bias vector of *i*_*t*_ are represented by *w*_*i*_ and *b*_*i*_, respectively. The sigmoid activation function, denoted by *σ*, is applied. Subsequently, $\hat{c_{t}}$ is introduced to represent the current input state. $\hat{c_{t}}$ is obtained by linearly transforming and applying the tanh activation function to the concatenation of the current input *x*_*t*_ and the previous hidden state *h*_*t*−1_. In other words, the state information contained in *x*_*t*_ and *h*_*t*−1_ is integrated to form a new state representation. $\hat{c_{t}}$ and the previous cell state *c*_*t*−1_ are used to calculate and update the current cell state *c*_*t*_. The specific formulas for the aforementioned process are as follows.

$$\hat{c_{t}}=\tau (w_{c}*[h_{t-1},x_{t}]+b_{c})$$ (3)

$$c_{t}=c_{t-1}*f_{t}+\hat{c_{t}}*i_{t}$$ (4)

Where the weight matrix and bias vector of $\hat{c_{t}}$ are denoted by *w*_*c*_ and *b*_*c*_, respectively. The symbol *τ* represents the tanh activation function. The calculation process of *o*_*t*_ is similar to that of *f*_*t*_ and *i*_*t*_. Additionally, the current hidden state *h*_*t*_ is determined by *c*_*t*_ and *o*_*t*_, and their computation formulas are as follows.

$$o_{t}=\sigma (w_{o}*[h_{t-1},x_{t}]+b_{o})$$ (5)

$$h_{t}=o_{t}*\tau (c_{t})$$ (6)

Where the weight matrix and bias vector of *o*_*t*_ are denoted by *w*_*o*_ and *b*_*o*_, respectively. The symbol *σ* represents the sigmoid activation function, and *τ* represents the tanh activation function.

LSTM addresses the issue of vanishing gradients during training by incorporating a series of gate mechanisms. However, as LSTM only propagates information in one direction, it can only learn forward features and not capture backward features. To overcome this limitation, Graves et al. introduced BiLSTM based on LSTM [33]. BiLSTM effectively combines a pair of forward and backward LSTM sequences, inheriting the advantages of LSTM while addressing the unidirectional learning problem. This integration allows BiLSTM to effectively capture contextual information in sequential data. From a temporal perspective, BiLSTM analyzes both the "past-to-future" and "future-to-past" directions of data flow, enabling better exploration of temporal features in the data and improving the utilization efficiency of the data and the predictive accuracy of the model.

**Fig 2:**
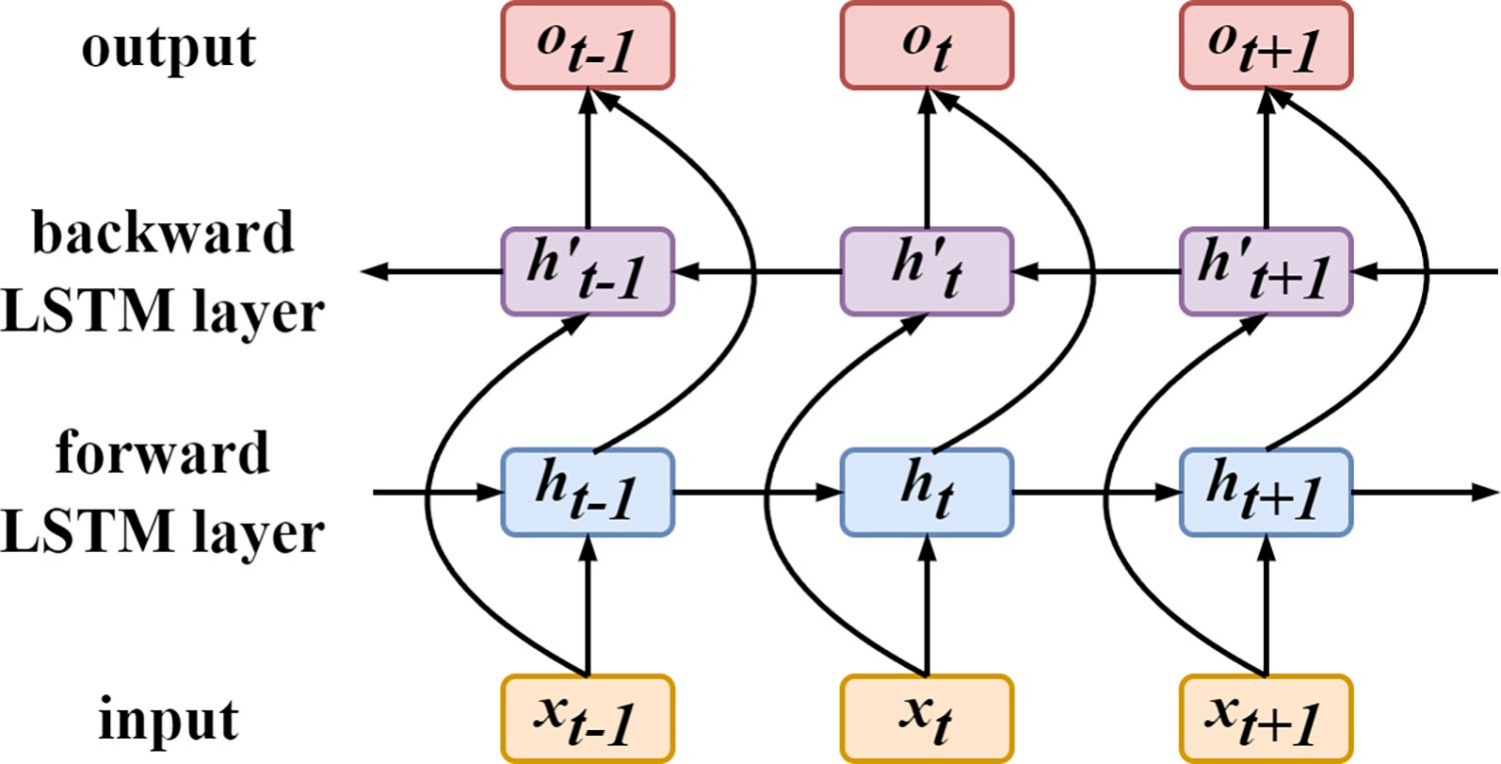
Schematic diagram of the BiLTSM.

Fig 2 depicts the unfolding of the BiLSTM network along the time axis, including *t*−1, *t*, and *t*+1. In the diagram, ***x*** represents the input to the model, ***h*** represents the hidden layer states, and ***o*** represents the output. BiLSTM can handle both forward and backward temporal information, thus having a hidden layer for each direction without mutual influence. The sequential data are separately fed into the forward LSTM layer and the backward LSTM layer, resulting in the computation of the hidden state *h*_*t*_ in the forward LSTM layer and the hidden state $h_{t}^{\prime }$ in the backward LSTM layer. It is important to note that the forward LSTM layer performs forward computation (from time 1 to time *t*), while the backward LSTM layer performs backward computation (from time *t* to time 1). The forward hidden states $\vec{h_{t}}$ and backward hidden states $\overset{\leftarrow }{h_{t}}$ for each time step are computed and stored accordingly. These two sets of hidden state information are further processed to generate the output of the BiLSTM. The process described above can be expressed using the following formulas.


$$\vec{h_{t}}=LSTM(x_{t},\vec{h_{t-1}})$$ (7)

$$\overset{\leftarrow }{h_{t}}=LSTM(x_{t},\overset{\leftarrow }{h_{t+1}})$$ (8)

$$o_{t}=W_{\vec{h}}*\vec{h_{t}}+W_{\overset{\leftarrow }{h}}*\overset{\leftarrow }{h_{t}}$$ (9)

Here, *o*_*t*_ represents the output of the BiLSTM. LSTM denotes the LSTM unit, $\vec{h_{t}}$ represents the output of the forward LSTM layer, and $\overset{\leftarrow }{h_{t}}$ represents the output of the backward LSTM layer. $W_{\vec{h}}$ and $W_{\overset{\leftarrow }{h}}$ are the weight matrix for $\vec{h_{t}}$ and $\overset{\leftarrow }{h_{t}}$, respectively.

### 3.3. Attention mechanism (AM)

It is now understood that human vision has the ability to rapidly locate key areas in images or text and focus attention on those regions to extract essential information. Inspired by human visual attention, a structure called the attention mechanism has been proposed and widely applied in fields such as machine translation [34] and gesture prediction [35]. The attention mechanism enhances important information in the input sequence while disregarding irrelevant details, thus optimizing the performance of the basic network. Specifically, the calculation of attention weights involves three steps. First, the score of the input vector *h*_*t*_ is computed, as shown in Formula 10.

$$s_{t}=\tau (w_{h}*h_{t}+b_{h})$$ (10)

Where *s*_*t*_ represents the attention score, *h*_*t*_ represents the input vector. *w*_*h*_ and *b*_*h*_ represent the weight matrix and bias matrix of the attention mechanism, respectively, and *τ* represents the tanh activation function. Next, *s*_*t*_ is normalized and the attention weights *a*_*t*_ can be obtained using the softmax function, which can be expressed as follows:

$$a_{t}=\frac{exp(s_{t})}{\sum_{t}exp(s_{t})}$$ (11)

Finally, the weighted sum of *a*_*t*_ and *h*_*t*_ is computed to obtain the final output enhanced by the attention mechanism.


$$s=\sum_{t}a_{t}h_{t}$$ (12)

### 3.4. Attention-based CNN-BiLSTM network (A-CBLN)

**Fig 3:**
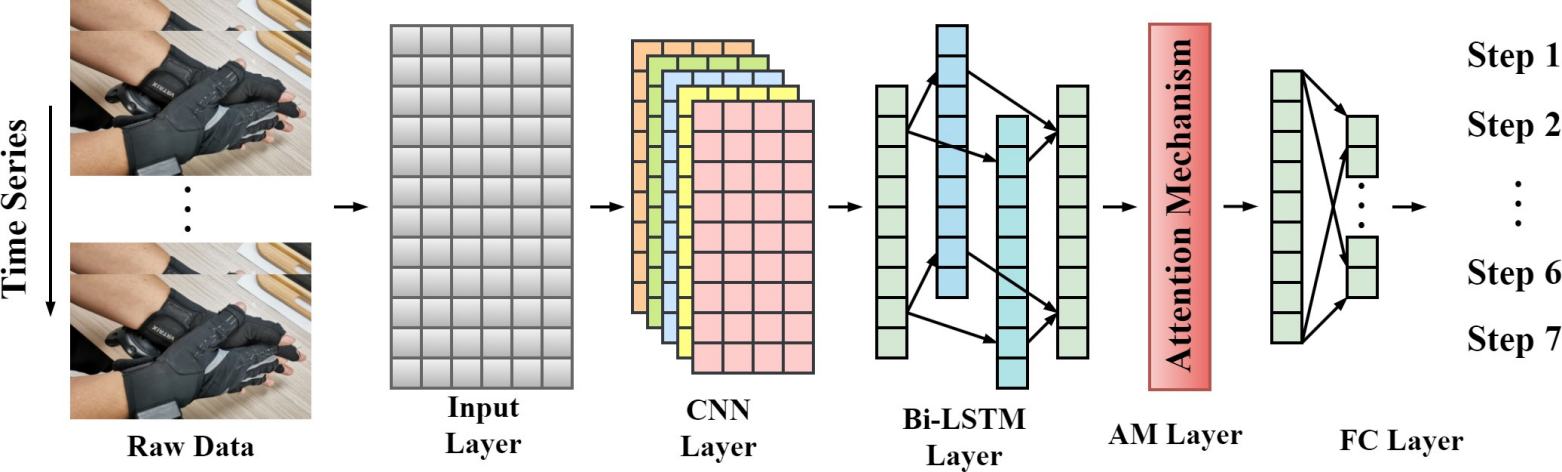
Dynamic gesture recognition pipeline based on A-CBLN.

This study aims to recognize the meaning conveyed by dynamic gesture data over time, which can be understood as a classification task for time series data. Building upon the previous discussions, it is highly conceivable that CNN can effectively extract local features from time series data, but may not capture long-range dependencies present in the data. The advantages of BiLSTM can overcome this limitation by learning from the forward and backward processes of dynamic gesture data, allowing the model to effectively capture the underlying long-term dependencies. Furthermore, the incorporation of attention mechanism can enhance the model’s semantic understanding of various gestures, thereby boosting the accuracy of gesture recognition. Therefore, in this paper, we proposed to combine CNN, BiLSTM, and the attention mechanism, presenting a novel framework for dynamic gesture recognition called Attention-based CNN-BiLSTM Network (A-CBLN). A-CBLN effectively integrates the advantages of different types of neural networks, thereby improving the predictive accuracy of dynamic gesture recognition. Fig 3 illustrates the pipeline of dynamic gesture recognition based on A-CBLN.

Specifically, as shown in Fig 3, the A-CBLN consists of five main components. The input layer transforms the data collected by the data glove into the model’s input format: *T*×*L*×1, where T represents the number of samples of gesture data collected within a specified time range, L represents the feature dimension of the gesture data returned by the data glove, and 1 represents the number of channels. The CNN layer performs feature extraction and dimensionality reduction using two convolutional operations and one max-pooling operation. It is worth noting that we did not directly employ 1D or 2D convolutional methods for feature extraction, but instead utilized a 2D convolutional method with a kernel size of 1×3, enabling the extraction of spatial features from gesture data without being influenced by the temporal dimension. The BiLSTM layer provides additional modeling of the long-term dependencies of gesture features. Both the CNN layer and the BiLSTM layer use the ReLU activation function. The AM layer helps the network better understand the specific meaning of gesture features. The FC layer utilizes fully connected layers to flatten the features and further reduce the dimensionality. Finally, it outputs the probability prediction of the current dynamic gesture through the softmax function. Table 1 presents the specific parameter settings for each network layer in A-CBLN.

**Table 1:** Specific parameter settings for each layer in A-CBLN.

Layer	Operation	Parameter Description
Input	Input	--
	Conv2D	kernel size = 1×3, filters = 16, strides = 1, activation = relu
CNN	Conv2D	kernel size = 1×3, filters = 32, strides = 1, activation = relu
	MaxPooling2D	kernel size = 1×2, strides = 2
BiLSTM	BiLSTM	neurons = 8
	BiLSTM	neurons = 8
AM	Attention	--
FC	Dense	neurons = 16, activation = relu
	Dense	neurons = 7, activation = softmax

We summarized the training and validation process of the A-CBLN algorithm in Algorithm 1. The two inputs of the algorithm include gesture data ***X*** and gesture label ***y***, and the output is the trained model weight w*. The predefined main parameters include epoch, batch size, and best validation accuracy. In line 1, The gesture dataset is divided into a training dataset and a validation dataset according to a certain proportion. In line 2, A-CBLN model randomly initializes parameters according to the default method. In line 3, the model begins the training and validation process. For each epoch, we first feed the training dataset into the model in sequence according to a certain batch (training data is ***X***_*train*_, training label is ***y***_*train*_). Next, we follow the feature extraction process of A-CBLN to extract features from ***X***_*train*_ and finally generate the corresponding prediction probability ${\hat{y}}_{train}$ (lines 6 to 9). In line 10, the cross-entropy loss function is used to calculate the loss value between ***y***_*train*_ and ${\hat{y}}_{train}$ and the Adam optimizer is used to update the parameters. After the training process is completed, we start the validation process, calculate the accuracy of the current model on the validation dataset and record it as *V*_*acc*_ (line 11). Then we determine the value of the current *V*_*acc*_ and the best validation accuracy. If *V*_*acc*_ is larger, then save the currently trained model parameters w* and update the value of the best validation accuracy to *V*_*acc*_ (Lines 12–14). After completing all training epochs, the entire training and validation process of A-CBLN ends (line 15).

**Algorithm 1. The pseudocode of A-CBLN**.

**Input**: Gesture Dataset ***X***, Gesture Labels ***y***

**Output**: Trained model weights: w*

**Parameter**:

Epoch: 50

Batch size: 64

Best validation accuracy: 0

1. Load training dataset and validation dataset from ***X* and y**;

2. Randomly initialize weight ***w***;

3. Start Training and Valid;

4. **For** each epoch **do**:

5.   **For** each batch (***X***_*train*_, ***y***_*train*_*)* in training dataset **do**:

6.     *F*_1_ is obtained by using two convolution layers on *X*_*train*_;

7.     *F*_2_ is obtained by using a max-pooling layer on *F*_1_;

8.     *F*_3_ is obtained by using two BiLSTM layers on *F*_2_;

9.     Using two FC layers on *F*_3_ to generate ${\hat{y}}_{train}$;

10.     Update weights of the model using the categorical cross-entropy loss function with the Adam optimizer;

11. Calculate the accuracy of the model on the validation dataset denoted as *V*_*acc*_;

12. **If** *V*_*acc*_ **>** Best Validation accuracy:

13.     Save Trained model weights w*

14.     Update the value of Best Validation accuracy to the value of *V*_*acc*_

15.**End** Training and Valid

## 4. Experiments

### 4.1. Data glove

The wearable sensor gesture data extraction device used in this study is provided by the VRTRIXTM Data Glove(http://www.vrtrix.com.cn/). The core component of this glove is a 9-axis MEMS (Micro Electro Mechanical System) inertial sensor, which can capture real-time motion data related to finger joints and enable the reproduction of the hand postures assumed by the operator during motion execution. The transmission of data from the glove employs wireless transmission technology, where the data captured by the sensors on both hands can be wirelessly transmitted to a personal computer (PC) through the wireless transmission module on the back of the hand for real-time rendering. In addition, the VRTRIXTM Data Glove provides a low-level Python API interface, allowing users to access joint pose data of the data glove, facilitating secondary development. It has been widely used in fields such as industrial simulation, mechanical control, and scientific research data acquisition.

Once the data glove is properly worn, the left hand has a total of 11 inertial sensors for capturing finger gestures. Specifically, each finger is assigned 2 sensors, while 1 sensor is allocated to the back of the hand. The number and distribution of sensors on the right hand are identical to those on the left hand. Table 2 presents the key parameters information of the data glove used in this study.

**Table 2:** Key parameters of the data glove used in this paper.

Parameter Name	Parameter details
Operating system	Win7 and above Windows operating system
Sensor module	A single hand is equipped with 11 high-precision, zero-drift nine-axis inertial sensor modules.
Product size	23cm × 12cm × 6cm
Maximum acceleration	±16g
Degrees of freedom	Three degrees of freedom (heading, traverse, pitch)
Angle range	360° for heading, traverse and pitch
Angle resolution	0.01°

### 4.2. Gesture definition

This study sought to explore the applications of dynamic gesture recognition in the field of medical virtual simulation based on wearable devices (data gloves). We first comprehensively reviewed the existing literature on dynamic gesture recognition. As mentioned in Section 2, most publicly available dynamic gesture datasets are based on visual systems, with only a few studies utilizing wearable devices. Therefore, we created a new dynamic gesture dataset based on the common seven-step handwashing in medical virtual simulation systems in conjunction with the data gloves. We followed the handwashing method recommended by the World Health Organization (WHO) [36] and established a complete handwashing procedure comprising seven steps. More details on these steps are presented in Table 3.

**Table 3:** Detailed description of the seven dynamic gestures.

Steps	Action details description
Step 1	The palms of the left and right hands face each other, with fingers closed together and rubbing against each other.
Step 2	The palm of the left hand is placed on the back of the right hand, with interlocked fingers, rubbing against each other. The motion is performed alternately with both hands.
Step 3	The palms of the left and right hands face each other, with fingers of both hands interlaced, rubbing against each other.
Step 4	The left hand grips the thumb of the right hand and rotates, rubbing and kneading. The motion is performed alternately with both hands.
Step 5	Bend the fingers of the left hand, rotating the joints, and rub them against the palm of the right hand. Perform this motion alternately with both hands.
Step 6	Bring the fingertips of the left hand together, pressing them against the palm of the right hand, and rotate while rubbing. Repeat this motion alternately with both hands.
Step 7	Place the palm of the left hand against the wrist of the right hand and rotate while rubbing, moving across the arm towards the elbow. Repeat this motion alternately with both hands.

### 4.3. Data acquisition and preprocessing

According to the approval of the Medical Ethics Committee of Affiliated Hospital of Xuzhou Medical University, 32 healthy subjects were recruited for this study. We organized and conducted data acquisition for this study between January 5, 2023 and March 25, 2023. Prior to gesture data collection, each subject was required to sign a consent form granting permission for their data to be used in the study and was informed of the specific steps involved in data collection. In order to ensure the precise expression of gesture actions while wearing the data gloves, participants who were initially unacquainted with the seven-step handwashing received training sessions conducted by healthcare professionals until all subjects could correctly perform the hand gestures while wearing the gloves. Additionally, a timekeeper was assigned to prompt the start and end of each gesture action and record the corresponding time information.

Once the subject correctly wore the data gloves as instructed, the gesture data collection process followed the following detailed steps:

1. Subjects kept their hands in the initial position, with both hands on the same horizontal plane, palms facing upward, and not more than 20cm apart.
2. The timekeeper issued the instruction to start the action, recorded the current time, and subjects began repeatedly performing the current gesture action within a 15-second interval. After 15 seconds, the timekeeper instructed to end the action and recorded the end time.
3. Subjects returned their hands to the initial position and prepared to collect data for the next gesture action following the same procedure as in step 2.

**Fig 4:**
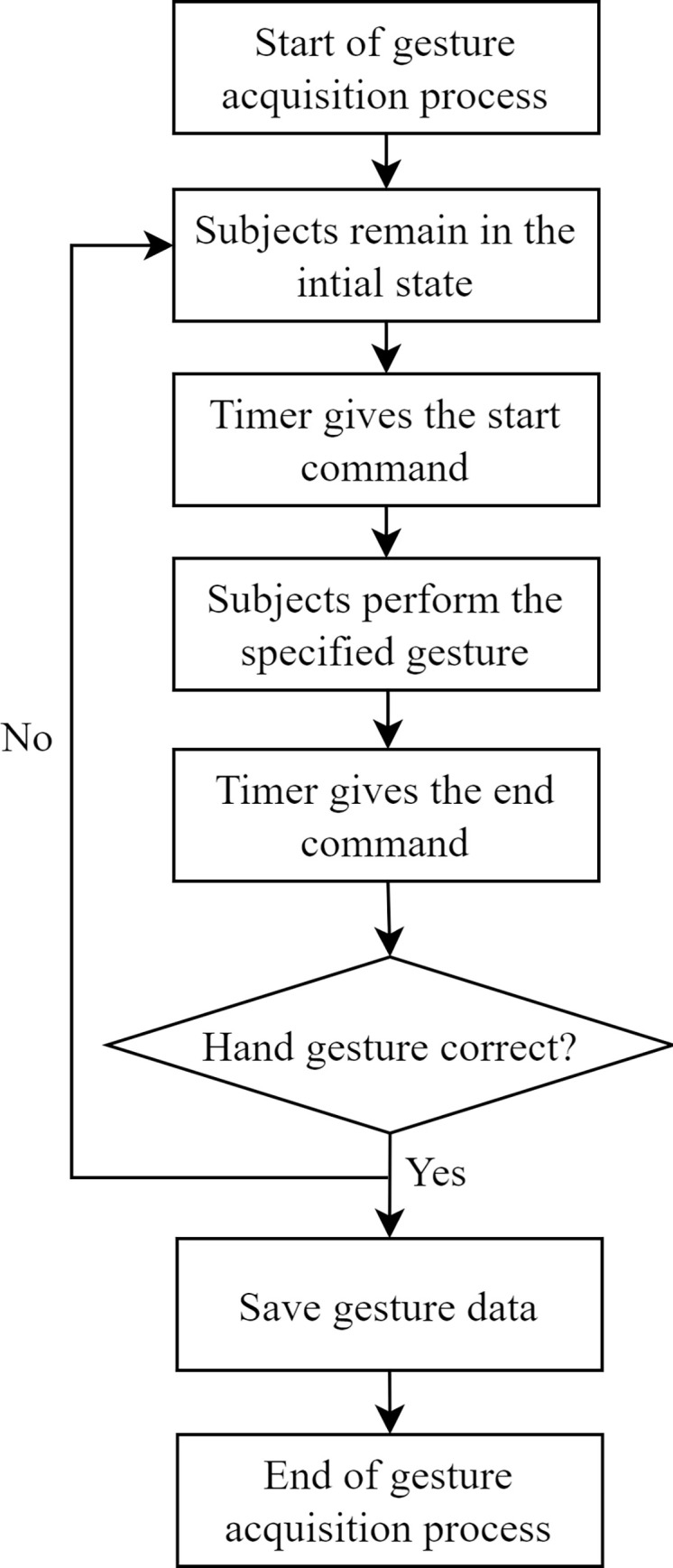
Flow chart of the gesture data acquisition.

Fig 4 illustrates the specific flow of gesture data acquisition. The data gloves used in this study provided a Python API interface, facilitating the recording of gesture data using Python scripts. The data for each subject were stored in individual folders named after their respective names. Additionally, subjects were requested to repeat the gesture collection process five times to increase the dataset size. Once data collection from all subjects was completed, the data were exported for further processing and analysis.

Specifically, the archival structure for each subject encompassed a set of five folders, and each folder consisted of seven dynamic gesture data files in text format. The data sampling frequency was set at 60Hz. We used a 3s time window to slide and segment the 15s data of each sample without overlapping, since the actions within 3s already contained the specific semantics of the current gesture. In summary, the sample size used for dynamic gesture modeling analysis in this study was 5,600, with each sample having a data dimension of (180×128×1). Here, 180 represents the number of gesture samples within 3 seconds, 128 represents the joint data returned by the data glove sensors, and 1 denotes the number of channels. Finally, to enhance the training of the gesture recognition model, a min-max scaling technique was applied to rescale the data intensity of all samples to the range [0, 1] using Formula 13.


$$f_{norm}=\frac{f-f_{min}}{f_{max}-f_{min}}$$ (13)

Here, *f* represents the input data, *f*_*norm*_ refers to the normalized data, *f*_*min*_ and *f*_*max*_ represent the minimum and maximum values of the input data, respectively.

To evaluate the performance of the proposed gesture recognition model, we divided the data into training, validation, and test dataset in a ratio of 8:1:1. Therefore, the data from 26 subjects were used for training, while the remaining 6 were evenly split between the validation and test dataset.

### 4.4. Implementation details

To validate the effectiveness of the proposed dynamic gesture recognition algorithm, we selected three deep learning algorithms related to gesture recognition research for comparison:

1. LSTM [37]: The model consists of 1 LSTM structure with 3 fully connected layers.
2. Attention-BiLSTM [25]: The model consists of two BiLSTM layers, an attention mechanism layer and a softmax classifier.
3. CNN-LSTM [38]: The model consists of a mixture of 2D convolutional layers, LSTM layers and fully connected layers.

All the experimental code in this study was written using Python (version 3.8). The deep learning algorithms were implemented using the TensorFlow framework (version 2.9.0). To ensure a fair comparison of the performance of each deep learning algorithm, we used the following training parameters consistently during the model training process: an initial training epoch of 50 and a batch size of 64. Since the research in this paper involved a typical multi-class classification task, we employed the cross-entropy loss to measure the error between the predicted values of the model and the true labels. We used the ADAM optimizer [37] to update the model parameters, and the initial learning rate was set to 0.001, the beta1 was set to 0.9, the beta2 was set to 0.999. After each training epoch, validation was performed, and the model with the lowest validation loss was saved for subsequent algorithm testing.

### 4.5. Evaluation metrics

This study employed four commonly used evaluation metrics in classification tasks to evaluate the gesture recognition performance of each model: accuracy (ACC), precision (Pr), recall (Re), and F1-score (F1). ACC represents the ratio of the number of correctly predicted gestures to the total number of evaluated samples. Pr is used to calculate the ratio between the number of correctly recognized positive samples and the total number of samples recognized as positive. Re measures the ratio between the number of correctly recognized positive samples and the total number of positive samples in all evaluated samples. F1 is computed based on Pr and Re, taking into account the balance between them. The formulas for the above metrics are shown below:

$$ACC=\frac{TP+TN}{TP+FP+TN+FN}$$ (14)

$$Pr=\frac{TP}{TP+FP}$$ (15)

$$Re=\frac{TP}{TP+FN}$$ (16)

$$F1=\frac{2\times Pr\times Re}{Pr+Re}$$ (17)

Here, TP represents the number of true positive samples, which are the samples that are correctly predicted as positive. TN represents the number of true negative samples, which are the samples that are correctly predicted as negative. FP represents the number of false positive samples, which are the samples that are actually negative but predicted as positive. FN represents the number of false negative samples, which are the samples that are actually positive but predicted as negative.

## 5. Results and analysis

This section presents and analyzes the effectiveness of all models for dynamic gesture recognition from multiple perspectives. It includes a comparative analysis of the learning capabilities of different models and their predictive performance on the test dataset. Additionally, we conducted relevant experiments to discuss the impact of key parameters in A-CBLN, including the kernel size in the convolutional layer and the number of neurons in the BiLSTM layer, the findings from these experiments provide valuable insights into the optimal configuration of A-CBLN for enhanced gesture recognition performance. Finally, we further analyzed and discussed the confusion matrix predicted by A-CBLN on the test dataset.

### 5.1. Comparative analysis between different models

**Fig 5:**
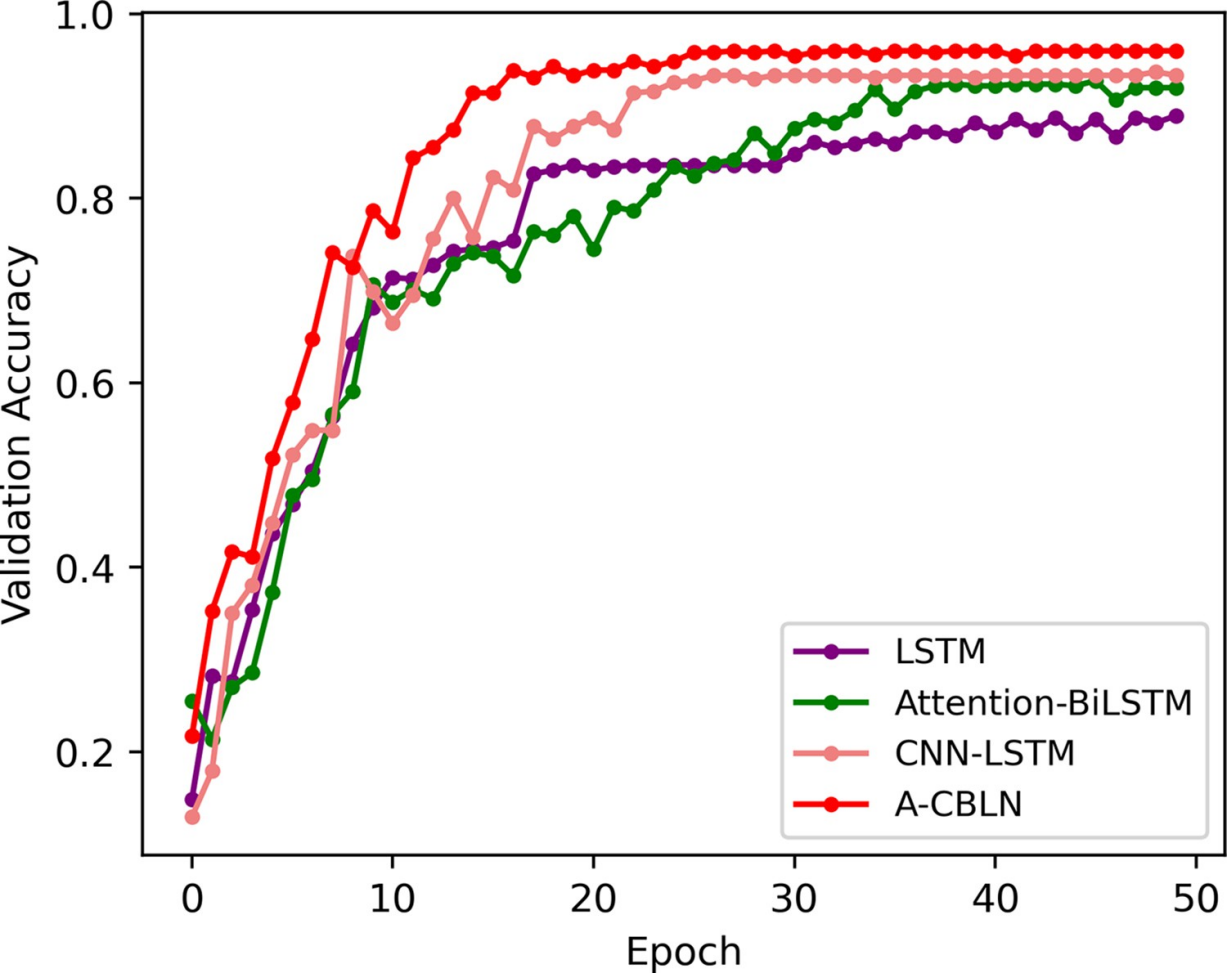
Variation process of validation accuracy of different models.

We first analyzed the learning progress of the models during the training process. Fig 5 shows that as the number of training epochs increases, the validation accuracy gradually improves and stabilizes for all models. This finding indicates that all models possess certain learning capabilities, and overfitting phenomena does not occur during training. Further analysis revealed that the single LSTM structure exhibits the lowest learning capability, reaching its highest validation accuracy of 88.95% at 50 epochs. This may be due to the fact that the pure LSTM structure fails to focus on the local features within the dynamic handwashing steps. For instance, actions like rubbing or rotating are of utmost importance in understanding the semantic meaning conveyed by the gestures. In contrast, the best validation accuracy of the Attention-BiLSTM structure has been improved and peaked at 45 epochs (92.77%). Nevertheless, the entire training progress displays instability. This limitation is also attributed to the structure’s limited ability in capturing local features. By combining CNN and LSTM, the model can not only perceive the local features of dynamic gestures in spatial changes but also capture their temporal variations. As a result, the recognition ability has been significantly improved, the model achieves an accuracy of 93.71% at 48 epochs. Finally, our proposed A-CBLN combines attention mechanisms, further enhancing the model’s understanding of different gesture semantics. Consequently, it exhibits the most powerful learning capability during the training process. Its validation accuracy stabilizes and consistently outperforms other models after 18 epochs, peaked at 32 epochs (93.62%).

The model with the best performance on the validation dataset was preserved for further analysis of their performance on the test dataset. As shown in Table 4, all models perform well on the test dataset, with prediction accuracy exceeding 87%. Further observation reveals that the pure LSTM and Attention-BiLSTM models have relatively lower prediction accuracy (87.43% and 91.43% respectively), while the hybrid CNN-LSTM structure significantly improves the prediction accuracy to 93.38%. This is consistent with our previous analysis, indicating that the hybrid CNN-LSTM structure possesses stronger feature extraction capability for dynamic gesture data. Finally, our proposed A-CBLN model demonstrates the best predictive performance for dynamic gestures, achieving optimal values in all evaluation metrics, with an accuracy of 95.05%, precision of 95.43%, recall of 95.25%, and F1-score of 95.22%. Compared to the pure LSTM structure, it improves by 7.62%, 5.84%, 7.32%, and 7.78% in accuracy, precision, recall, and F1-score, respectively.

**Table 4:** Prediction results of different models on the test dataset.

Model	ACC (%)	Pr (%)	Re (%)	F1 (%)
LSTM	87.43	89.59	87.93	87.44
Attention-BiLSTM	91.43	93.00	91.98	91.40
CNN-LSTM	93.33	93.70	93.58	93.52
A-CBLN	95.05	95.43	95.25	95.22

### 5.2. Different size of convolution kernels of the A-CBLN

**Fig 6:**
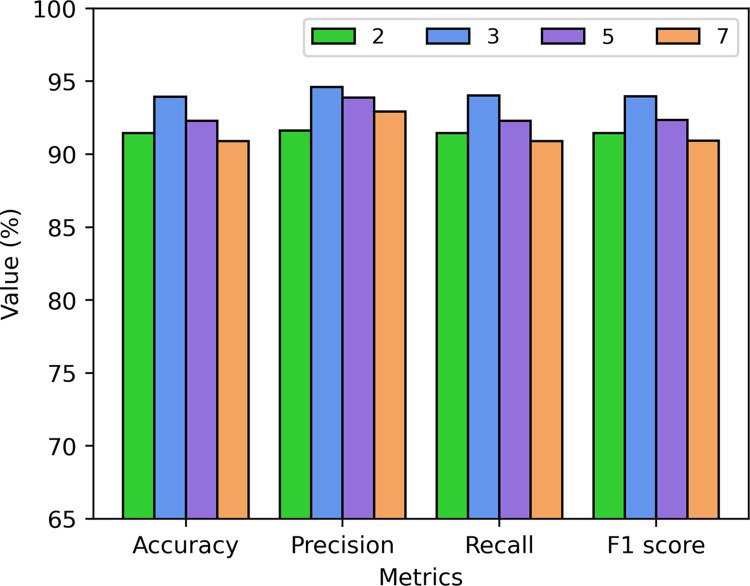
Effect of different convolutional kernel size on the A-CBLN algorithm.

The choice of different kernel size in convolutional layers implies variations in the receptive field for extracting local features. Therefore, selecting an appropriate kernel size is crucial for improving model performance. We conducted a comparative analysis to investigate the impact of four different kernel sizes (1×2, 1×3, 1×5, and 1×7) on the recognition performance of the A-CBLN algorithm. Fig 6 reveals that the recognition performance of the A-CBLN algorithm initially improves and then declines as the kernel size increases. Through further observation, it can be noted that the utilization of large convolutional kernels can lead to a decrease in the overall recognition performance of the model. This is because, while enlarging the receptive field, they also extract redundant features. When the kernel size is set to 1×3, the A-CBLN algorithm achieves the optimal performance in terms of accuracy, precision, recall, and F1- score. The corresponding performance metrics reach their peak values of 93.94%, 94.60%, 94.02%, and 93.98%, respectively.

### 5.3. Number of neurons in BiLSTM of the A-CBLN

**Fig 7:**
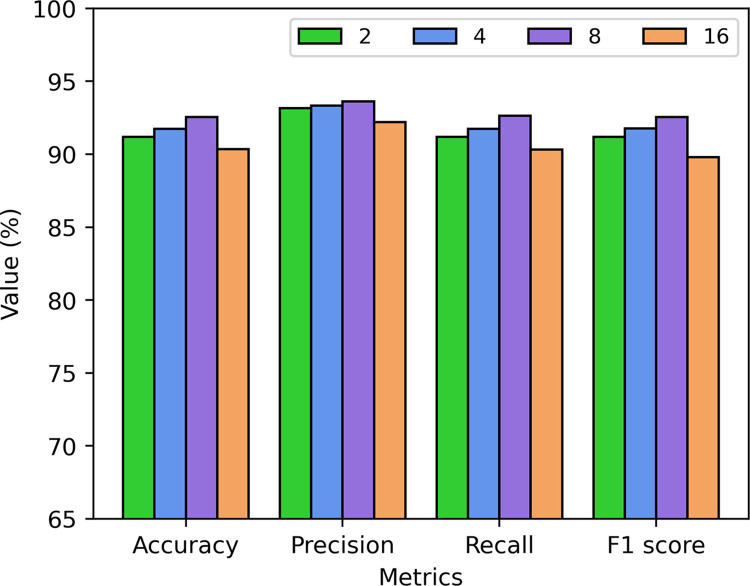
Effect of different number of neurons in BiLSTM on A-CBLN algorithm.

The number of neurons in the BiLSTM layer also influences the recognition performance of the A-CBLN algorithm. In this section, we discussed four different neuron quantities: 2, 4, 8, and 16. As shown in Fig 7, the recognition performance of the A-CBLN algorithm initially improves and then declines with an increase in the number of neurons in the BiLSTM layer. When the neuron quantity is set to 8, the A-CBLN algorithm achieves the optimal performance in terms of accuracy, precision, recall, and F1-score. The corresponding performance metrics reach their peak values of 92.56%, 93.63%, 92.64%, and 92.54%, respectively.

### 5.4. Confusion matrix of the A-CBLN on the test dataset

**Fig 8:**
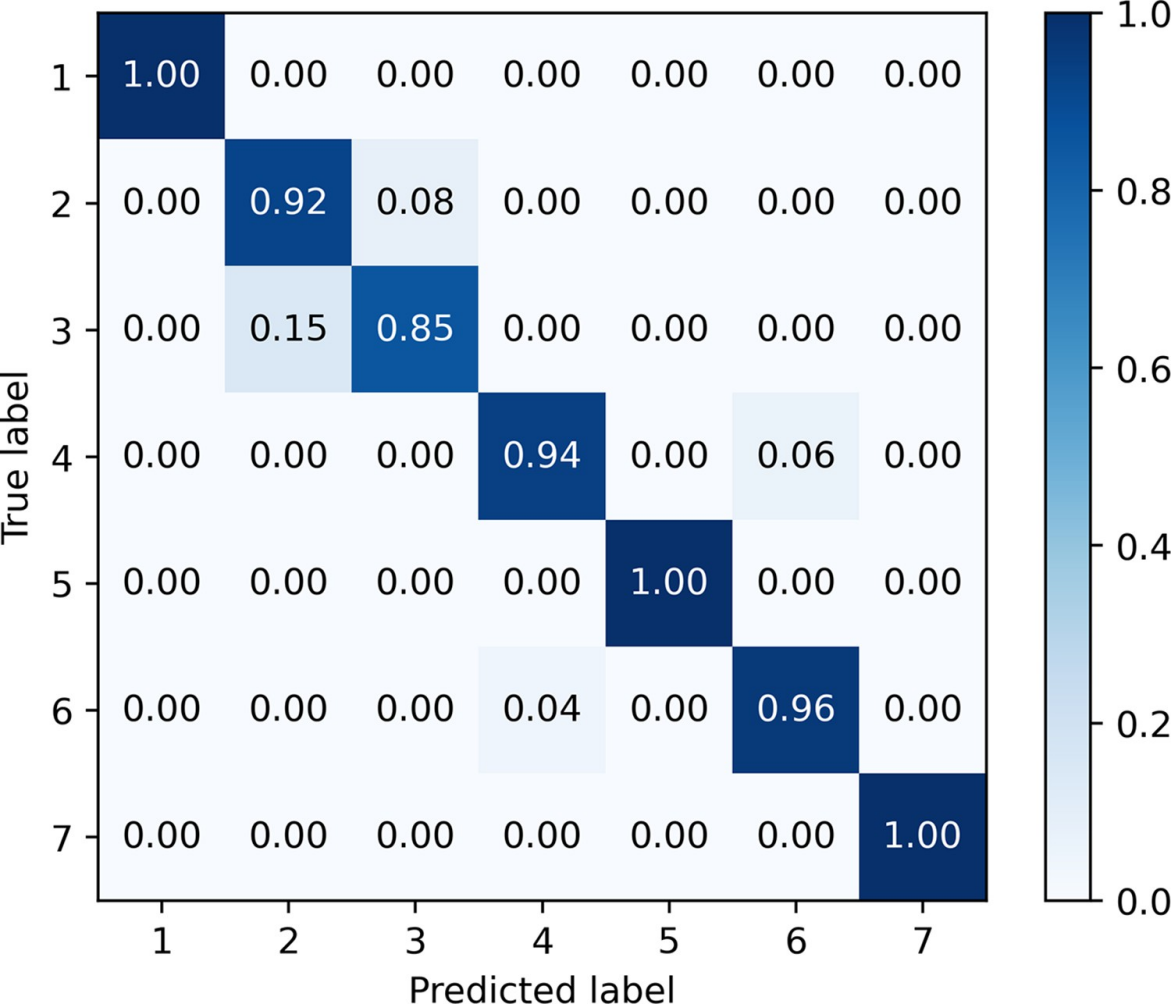
Confusion matrix of A-CBLN on the test dataset.

Finally, we conducted a separate discussion and analysis of the prediction results of the A-CBLN algorithm on the test dataset. As shown in Fig 8, the values on the main diagonal of the confusion matrix represent the percentage of correctly predicted samples in each gesture category, while the remaining positions indicate cases where the model incorrectly predicts a given gesture as another category. Upon further observation, it can be determined that A-CBLN achieves recognition accuracy higher than 85% for all seven handwashing steps. Specifically, the model achieves perfect recognition for gestures in steps 1, 5, and 7, as these gestures exhibit distinct spatial features. However, the recognition performance for handwashing step 3 actions is poor, with approximately 15% of the samples incorrectly classified as step 2. This may be attributed to the similarity between the hand gestures in these two steps, involving actions such as "finger crossing" and "mutual friction," which the two convolutional layers in A-CBLN may struggle to differentiate between them. Additionally, there are also some recognition errors for handwashing actions in steps 4 and 6, likely due to the presence of similar actions such as "finger bending" and "rotational friction," leading to misjudgment by the model. Overall, A-CBLN demonstrates good overall recognition performance for the seven dynamic gestures, with an average accuracy exceeding 95%.

## 6. Conclusion

This paper aims to investigate the problem of dynamic gesture recognition based on data gloves. Based on deep learning techniques, we proposed a dynamic gesture recognition algorithm called A-CBLN, which combines structures such as CNN, BiLSTM, and attention mechanism to capture the spatiotemporal features of dynamic gestures to the maximum extent. We selected the commonly used seven-step handwashing method in the medical simulation domain as the research subject and validated the performance of the proposed model in recognizing the seven dynamic gestures. The experimental results demonstrated that our proposed approach effectively addresses the task of dynamic gesture recognition and achieved superior prediction results compared to similar models, with the accuracy of 95.05%, precision of 95.43%, recall of 95.25%, and F1-score of 95.22% on the test dataset. In the future, we plan to further improve our approach in the following aspects: (1) design more efficient feature extraction modules to enhance the discriminability of gestures with similar action sequences; (2) recruit more subjects to increase the dataset size and improve the model’s generalization ability; (3) explore the fusion of multimodal data captured by infrared cameras to enhance the recognition performance of the model.

## References

[1] Shah A, Ali B, Habib M, Frnda J, Ullah I, Shahid Anwar M. An ensemble face recognition mechanism based on three-way decisions. Journal of King Saud University—Computer and Information Sciences. 2023 Apr;35(4):196–208. doi: 10.1016/j.jksuci.2023.03.016

[2] Guo L, Lu Z, Yao L. Human-Machine Interaction Sensing Technology Based on Hand Gesture Recognition: A Review. IEEE Trans Human-Mach Syst. 2021 Aug;51(4):300–9. doi: 10.1109/THMS.2021.3086003

[3] LI Y, HUANG J, TIAN F, WANG H, DAI G. Gesture interaction in virtual reality. Virtual Reality & Intelligent Hardware. 2019 Feb;1(1):84–112. doi: 10.3724/SP.J.2096-5796.2018.0006

[4] Liu H, Wang L. Gesture recognition for human-robot collaboration: A review. International Journal of Industrial Ergonomics. 2018 Nov;68:355–67. doi: 10.1016/j.ergon.2017.02.004

[5] Peng Y, Wang J, Pang K, Liu W, Meng J, Li B. A Physiology-Based Flexible Strap Sensor for Gesture Recognition by Sensing Tendon Deformation. IEEE Sensors J. 2021 Apr 1;21(7):9449–56. doi: 10.1109/JSEN.2021.3054562

[6] Song X, Peng Y, Hu B, Liu W. Characterization of the fine hand movement in badminton by a smart glove. Instrumentation Science & Technology. 2020 Jul 3;48(4):443–58. doi: 10.1080/10739149.2020.1737814

[7] Zhang F, Chu S, Pan R, Ji N, Xi L. Double hand-gesture interaction for walk-through in VR environment. 2017 IEEE/ACIS 16th International Conference on Computer and Information Science (ICIS). 2017 May; doi: 10.1109/ICIS.2017.7960051

[8] Sun Y, Weng Y, Luo B, Li G, Tao B, Jiang D, et al. Gesture recognition algorithm based on multi‐scale feature fusion in RGB‐D images. IET Image Processing. 2023 Mar;17(4):1280–90. doi: 10.1049/ipr2.12712

[9] Kumar P, Gauba H, Pratim Roy P, Prosad Dogra D. A multimodal framework for sensor based sign language recognition. Neurocomputing. 2017 Oct;259:21–38. doi: 10.1016/j.neucom.2016.08.132

[10] Erol A, Bebis G, Nicolescu M, Boyle RD, Twombly X. Vision-based hand pose estimation: A review. Computer Vision and Image Understanding. 2007 Oct;108(1–2):52–73. doi: 10.1016/j.neucom.2016.08.132

[11] Sidig AaI, Luqman H, Mahmoud SA. Transform-based Arabic sign language recognition. Procedia Computer Science. 2017;117:2–9. doi: 10.1016/j.procs.2017.10.087

[12] Dong Y, Liu J, Yan W. Dynamic Hand Gesture Recognition Based on Signals From Specialized Data Glove and Deep Learning Algorithms. IEEE Trans Instrum Meas. 2021;70:1–14. doi: 10.1109/TIM.2021.307796733776080

[13] Karami A, Zanj B, Sarkaleh AK. Persian sign language (PSL) recognition using wavelet transform and neural networks. Expert Systems with Applications. 2011 Mar;38(3):2661–7. doi: 10.1016/j.eswa.2010.08.056

[14] Thalange A, Dixit S. COHST and Wavelet Features Based Static ASL Numbers Recognition. Procedia Computer Science. 2016;92:455–60. doi: 10.1016/j.procs.2016.07.367

[15] Wu C, Wang K, Cao Q, Fei F, Yang D, Lu X, et al. Development of a Low-Cost Wearable Data Glove for Capturing Finger Joint Angles. Micromachines. 2021 Jun 30;12(7):771. doi: 10.3390/mi12070771 34208871 PMC8304804

[16] Lee S, Choi Y, Sung M, Bae J, Choi Y. A Knitted Sensing Glove for Human Hand Postures Pattern Recognition. Sensors. 2021 Feb 15;21(4):1364. doi: 10.3390/s21041364 33671966 PMC7919032

[17] Antillon DWO, Walker CR, Rosset S, Anderson IA. Glove-Based Hand Gesture Recognition for Diver Communication. IEEE Trans Neural Netw Learning Syst. 2022; doi: 10.1109/TNNLS.2022.3161682 35439141

[18] Yuan X, Liang N, Fu W, Wang Q, Zhang Y, Cao J, et al. A Wearable Gesture Recognition System With Ultrahigh Accuracy and Robustness Enabled by the Synergy of Multiple Fabric Sensing Devices. IEEE Sensors J. 2023 May 15;23(10):10950–8. doi: 10.1109/JSEN.2023.3265775

[19] Ge Y, Li B, Yan W, Zhao Y. A real-time gesture prediction system using neural networks and multimodal fusion based on data glove. 2018 Tenth International Conference on Advanced Computational Intelligence (ICACI). 2018 Mar; doi: 10.1109/ICACI.2018.8377532

[20] Hoang N N, Lee G S, Kim S H, Yang H J. Continuous hand gesture spotting and classification using 3D finger joints information. 2019 IEEE International conference on image processing (ICIP). 2019 Sep; doi: 10.1109/ICIP.2019.8803813

[21] Plawiak P, Sosnicki T, Niedzwiecki M, Tabor Z, Rzecki K. Hand Body Language Gesture Recognition Based on Signals From Specialized Glove and Machine Learning Algorithms. IEEE Trans Ind Inf. 2016 Jun;12(3):1104–13. doi: 10.1109/TII.2016.2550528

[22] Ayodele E, Bao T, Zaidi SAR, Hayajneh AMA, Scott J, Zhang Z, et al. Grasp Classification With Weft Knit Data Glove Using a Convolutional Neural Network. IEEE Sensors J. 2021 May 1;21(9):10824–33. doi: 10.1109/JSEN.2021.3059028

[23] Lee M, Bae J. Deep Learning Based Real-Time Recognition of Dynamic Finger Gestures Using a Data Glove. IEEE Access. 2020;8:219923–33. doi: 10.1109/ACCESS.2020.3039401

[24] Yuan G, Liu X, Yan Q, Qiao S, Wang Z, Yuan L. Hand Gesture Recognition using Deep Feature Fusion Network based on Wearable Sensors. IEEE Sensors J. 2020; doi: 10.1109/JSEN.2020.3014276

[25] Wang H, Ru B, Miao X, Gao Q, Habib M, Liu L, et al. MEMS Devices-Based Hand Gesture Recognition via Wearable Computing. Micromachines. 2023 Apr 27;14(5):947. doi: 10.3390/mi14050947 37241571 PMC10223752

[26] Hu Y, Wong Y, Wei W, Du Y, Kankanhalli M, Geng W. A novel attention-based hybrid CNN-RNN architecture for sEMG-based gesture recognition. PLoS ONE. 2018 Oct 30;13(10):e0206049. doi: 10.1371/journal.pone.0206049 30376567 PMC6207326

[27] Lecun Y, Bottou L, Bengio Y, Haffner P. Gradient-based learning applied to document recognition. Proc IEEE. 1998;86(11):2278–324. doi: 10.1109/5.726791

[28] Li Y, Huang C, Ding L, Li Z, Pan Y, Gao X. Deep learning in bioinformatics: Introduction, application, and perspective in the big data era. Methods. 2019 Aug;166:4–21. doi: 10.1016/j.ymeth.2019.04.008 31022451

[29] Nair V, Hinton G E. Rectified linear units improve restricted boltzmann machines. Proceedings of the 27th international conference on machine learning (ICML-10). 2010: 807–814.

[30] Connor J, Martin R, Atlas L. Recurrent neural networks and robust time series prediction. IEEE Trans Neural Netw. 1994 Mar;5(2):240–54. doi: 10.1109/72.279188 18267794

[31] Shi H, Miao K, Ren X. Short‐term load forecasting based on CNN‐BiLSTM with Bayesian optimization and attention mechanism. Concurrency and Computation. 2023 Aug;35(17). doi: 10.1002/cpe.6676

[32] Greff K, Srivastava RK, Koutnik J, Steunebrink BR, Schmidhuber J. LSTM: A Search Space Odyssey. IEEE Trans Neural Netw Learning Syst. 2017 Oct;28(10):2222–32. doi: 10.1109/TNNLS.2016.2582924 27411231

[33] Graves A, Fernández S, Schmidhuber J. Bidirectional LSTM networks for improved phoneme classification and recognition. International conference on artificial neural networks. Berlin, Heidelberg: Springer Berlin Heidelberg, 2005: 799–804. doi: 10.1007/11550907_126

[34] Luong M T, Pham H, Manning C D. Effective Approaches to Attention-based Neural Machine Translation. Proceedings of the 2015 Conference on Empirical Methods in Natural Language Processing. 2015: 1412–1421. doi: 10.18653/v1/D15-1166

[35] Liu J, Wei B, Cai M, Xu Y. Dynamic gesture recognition based on CNN-LSTM-Attention. 2021 IEEE International Conference on Signal Processing, Communications and Computing (ICSPCC). IEEE, 2021: 1–6. doi: 10.1109/ICSPCC52875.2021.9565034

[36] Pittet D, Allegranzi B, Boyce J. The World Health Organization Guidelines on Hand Hygiene in Health Care and Their Consensus Recommendations. Infect Control Hosp Epidemiol. 2009 Jul;30(7):611–22. doi: 10.1086/600379 19508124

[37] Toro-Ossaba A, Jaramillo-Tigreros J, Tejada JC, Peña A, López-González A, Castanho RA. LSTM Recurrent Neural Network for Hand Gesture Recognition Using EMG Signals. Applied Sciences. 2022 Sep 27;12(19):9700. doi: 10.3390/app12199700

[38] Skaria S, Huang D, Al-Hourani A, Evans R J, Lech M. Deep-learning for hand-gesture recognition with simultaneous thermal and radar sensors. 2020 IEEE SENSORS. IEEE, 2020: 1–4. doi: 10.1109/SENSORS47125.2020.9278683

